# Data in support of the longitudinal characterization of pulmonary function in children with Mucopolysaccharidoses IVA

**DOI:** 10.1016/j.dib.2019.104756

**Published:** 2019-11-06

**Authors:** Johnny J. Kenth, Gabrielle Thompson, Stuart Wilkinson, Simon Jones, I.A. Bruce

**Affiliations:** a Department of Anesthesia, Royal Manchester Children's Hospital, Manchester University NHS Foundation Trust, Manchester, UK; b Manchester Academic Health Science Centre, The University of Manchester, Manchester, UK; c Pediatric ENT Department, Royal Manchester Children's Hospital, Manchester University NHS Foundation Trust, Manchester, UK; d Department of Pediatric Respiratory Medicine, Royal Manchester Children's Hospital, Manchester University NHS Foundation Trust, Manchester, UK; e Pediatric Inborn Errors of Metabolism, Willink Biochemical Genetics Unit, Manchester Centre for Genomic Medicine, St Mary's Hospital, Manchester University NHS Foundation Trust, Manchester, UK

**Keywords:** Morquio syndrome, Mucopolysaccharidosis IVA, MPS, Respiratory changes, Enzyme replacement therapy, Sleep-disordered breathing

## Abstract

Mucopolysaccharidoses type IVA (Morquio disease) is a rare, autosomal recessive lysosomal storage disease that causes both obstructive and restrictive airway pathology, with respiratory failure being the primary cause of death. This article provides original data on the longitudinal characterization of pulmonary function changes in children with Mucopolysaccharidoses (MPS) IVA by presenting the data and nuanced trends of changes from sequential spirometry and oximetry. The sample size included 16 subjects, 13 had undergone enzyme replacement therapy (ERT), three had not undergone ERT treatment. A total of 180 individual plots are presented for spirometry variables (FEV1, FEV1 [%Pred] FVC, FVC [%Pred] and FEV1/FVC), 6MWT and oximetry variables (median %Spo2, ODI 3%, mean nadir 3%, ODI 4%, mean nadir 4% and min dip SpO2 [%]); over a nine-year period at a single quaternary paediatric metabolic centre. This data has been made public and has utility to clinicians and researchers due to the following: [1,2] by providing the first comprehensive report of detailed changes in pulmonary function in children with MPS IVA, with and without ERT; [1–3] as well as changes in pulmonary function following the institution of non-invasive ventilation (NIV) and adenotonsillectomy. The data presented is related to the research article by Kenth et al. “The Characterization of Pulmonary Function in Patients with Mucopolysaccharidoses IVA: A Longitudinal Analysis”.

## Abbreviations

6MWT: 6-minute walk test
ADLs: Activities of daily living
AEs: Adverse Events
C6S: Chondroitin Sulphate
ERT: Enzyme replacement therapy
FEV_1_: Forced expiratory volume in one second
FEV_1_ [%Pred]: FEV1 as a percentage of predicted
FVC: Forced vital capacity
FVC: [%Pred]FVC as a percentage of predicted
GAG: Glycosaminoglycan
GALNS: Acetylgalactosamine-6-sulfatase
KS: Keratan sulphate
LSD: Lysosomal storage disease
MPS: Mucopolysaccharidosis IVA
Med nadir 3%: Median nadir of arterial oxygen saturations 3% from baseline
Min dip Spo2: Minimum dips in arterial oxygen saturations [%]
ODI 3%: Oxygen desaturation index; ≥ 3% arterial oxygen desaturations/hour

**Table:** Specifications Table

Subject area	*Biology, Medicine*
More specific subject area	*Inherited Metabolic Disorders*
Type of data	*Table, text file, graphs and figures*
How data was acquired	*From case notes, image archives and laboratory data to accrue baseline demographics, spirometry and oximetry measurements of MPS IVA patients.*
Data format	*Raw, filtered and analysed.*
Experimental factors	*Baseline demographics, longitudinal changes in spirometry and oximetry after enzyme replacement therapy; with mean* ± *SD, median (*25th–75th *percentile)*
Experimental features	*Longitudinal retrospective study*
Data source location	*The Royal Manchester Children's Hospital, Manchester. UK*
Data accessibility	*The data are accessible within the article* *Available from Mendeley* *Kenth, Johnny; Wilkinson, Stuart; Jones, Simon; Bruce, Iain(2019), “The Characterisation of Pulmonary Function in Patients with Mucopolysaccharidoses IVA: A Longitudinal Analysis.“, Mendeley Data, v3*https://doi.org/10.17632/wdsfn8wczc.3
Related research article	**Author's name:** **Title:** The Characterization of Pulmonary Function in Patients with Mucopolysaccharidoses IVA: A Longitudinal Analysis by Kenth et al. in Molecular Genetics and Metabolism, Elsevier (in press). **Journal:** Molecular Genetics and Metabolism Reports **DOI:**https://doi.org/10.1016/j.ymgmr.2019.100487

**Table:** 

**Value of the Data** • The data presented are the first comparison of enzyme replacement therapy (ERT) and non-ERT treated, in terms of the long-term decline in pulmonary function. • The data is grounded from longitudinal, standardized, repeated measurements taken from spirometry and oximetry studies. • The data is the first to report on the long-term changes in pulmonary function after an intervention such as non-invasive ventilation (NIV) and adenotonsillectomy. • These data are useful to clinicians and researchers evaluating the safety and efficacy of ERT on children with MPS IVA, especially within the context of evaluating airway respiratory changes.

### 1 Data

We present original data from a retrospective, longitudinal, repeated-measures cohort study, where descriptive statistics and non-parametric correlation were performed for demographic, respiratory function and oximetry (sleep studies) variables over a study period from January 2009 to December 2018. Composite clinical endpoints used in this study for evaluating pulmonary function included spirometry variables (FEV_1_, FEV_1_ [%Pred] FVC, FVC [%Pred] and FEV1/FVC), 6MWT and oximetry variables (median %Spo2, ODI 3%, mean nadir 3%, ODI 4%, mean nadir 4% and min dip SpO2 [%]). The data are supplemental to our study “The Characterization of Pulmonary Function in Patients with Mucopolysaccharidoses IVA: A Longitudinal Analysis” by Kenth et al. [1].

#### 1.1 Baseline demographics

Thirteen children underwent ERT, whereas the non-ERT arms had 3 children. The *median age* at diagnosis was 34 months (range: 14–161; IQR 62.75). The *Presenting symptoms* included: difficulty walking (7), gibbus deformity (4), kyphoscoliosis (1) and chest-wall deformity (1). *Consanguinity* of parents was present in 12.5 [2/16] of patients. The median age of commencing ERT was 78 months (IQR 77.5; 10th percentile 38.4, 90th percentile 179.8). The study included 44% of males (7/16) and 56% of females (9/16). The following patterns of genetic abnormalities were present (see Table 1), heterozygous p. (Gly155Arg), c.423-11_425del14/c.860C > T, heterozygous l113F, Y240C, heterozygous p. (l113F) and p. (R386H), heterozygous p. (arg251Ter), heterozygous p. (tyr254cys) and p. (Gln311Pro), heterozygous p. (w141×), heterozygous p. (His166Arg), heterozygous p. (A291T) with no discernible differences between the ERT treated and non-ERT treated groups. Table 1 illustrates the baseline demographics, including age at diagnosis, gender, parental consanguinity, what the presenting symptom was, the genetic mutation identified, the date when ERT therapy commenced (if applicable). Data are also presented on the age at first spirometry and oximetry, the presence of obstructive sleep apnoea, whether the patients had undergone an adenotonsillectomy and the institution on non-invasive, bi-level positive pressure ventilation (BIPAP).

**Table 1:** Baseline demographics.

Patient	Presentation	Age at Diagnosis^a^	Consanguinity		Sex		Genetics	Age ERT started^a^		Age at 1st spirometry^a^		Age at 1st oximetry^a^		OSA		Adeno-tonsillectomy		BIPAP	
ERT treated subjects																			
A	Difficulty walking	37	No		F		Heterozygous p.(Gly155Arg)	43		51		38		No		No		No	
B	Gibbus	27	No		M		Homozygous p.(A291T)	112		105		106		Yes		Yes (72, 147)		Yes	
C	Gibbus	22	N/A		M		Not recognised gene	39		71		39		Yes		(41)		No	
D	Difficulty walking	18	No		M		Homogenous p.(w141×)	78		91		88		Yes		No		Yes	
E	Difficulty walking, scoliosis	30	N/A		F		N/A	67		67		67		No		No		No	
F	Chest deformity	43	No		F		Heterozygous p.(arg251Ter)	69		124		74		Yes		Yes (121)		No	
G	N/A	132	No		M		Homozygous p.(A291T)	78		132		122		Yes		Yes (70, 142)		Yes	
H	N/A	35	N/A		F		c.423-11_425del14/c.860C > T	38				48		Yes		Yes (32, 51, 89)		No	
I	Family history, chest deformity	31	No		M		Hetero/l113F, Y240C	183		155		155		Yes		Yes (36)		No	
J	Difficulty walking	131	Yes		F		Homozygous p. (His166Arg)	175		141		133		Yes		No		No	
K	Gibbus	14	No		M		Heterogenous p. (l113F) and p. (R386H)	43		82		24		Yes		Yes (35)		No	
L	Difficulty walking	73	No		F		Heterozygous p.(tyr254cys) and p. (Gln311Pro)	108		118		105		No		No		No	
M	Gibbus, stiff joints	33	Yes		F		Homozygous p.(A291T)	129		101		100		No		Yes (121)		No	
Non ERT treated subjects																			
N	Difficulty walking	29	No	M		Homogenous p.(Ser264Asn)			N/A		102		31		Yes		No		Yes
O	Growth, skeletal dysplasia	96	N/A	F		Homogenous p. (Gly116Val)			N/A		102		98		Yes		Yes (134)		Yes
P	Difficulty walking	161	N/A	F		Heterozygous p.901G > T (Gly301Cys)			N/A		160		N/A		No		No		No

Table 1 above illustrates the baseline demographics of the 16 subjects, including age at diagnosis, consanguinity, what the presenting symptom was, the genetic mutation identified, the date when ERT therapy commenced (if applicable), the age at first spirometry test and the age at first oximetry. We also record whether the child was diagnosed with obstructive sleep apnoea (OSA), if they had undergone an adenotonsillectomy and the whether the child had been instituted on non-invasive, bilevel ventilation (BIPAP).

a All ages are reported in months.

Macroglossia was present in 44% of patients (7/16), video laryngoscopy was used in 68.8% [11/16] of cases. Mallampati score on airway assessment was as follows: grade 1 in 25% [4/16] of patients, grade 2a in 37.5% [6/16], grade 2b in 12.5% [2/16], grade 3 in 7.7% [1/16] and no grade recorded in 18.8% [3/16]. Clinical symptoms of OSA was present in 68.6% (11/16) of patients. 66.7% (10/15) of patients in the study had undergone an adenotonsillectomy. The median age of adenotonsillectomy was 80 (range: 35–147; IQR 87.3; 10th percentiles 35.4, 90th percentile, 145.5) with 3 patients had repeated procedures. 31.3% (5/16) of patients were on BIPAP; the median age of commencing BIPAP was 153 months (IQR 53). 75% (12/16) of patients had undergone orthopaedic interventions, this included: C-spine fixation and halo brace in 12.5% (2/16) and 31.3% (5/16) underwent hip replacement and epiphysiodesis (8-plate).

Cardiac disease, as assessed by echocardiography was normal in 62.5% (10/16) of patients, pathology delineated included, a small patent ductus arteriosus (PDA) in 6.3% (1/16), small PDA, mild tricuspid regurgitation (TR) in 6.3% (1/16), physiological mitral (MR) in 6.3% (1/16), physiological TR + MR in 6.3% (1/16), dysplastic mitral and aortic valve in 6.3% (1/16).

#### 1.2 Spirometry data

**Fig. 1:**
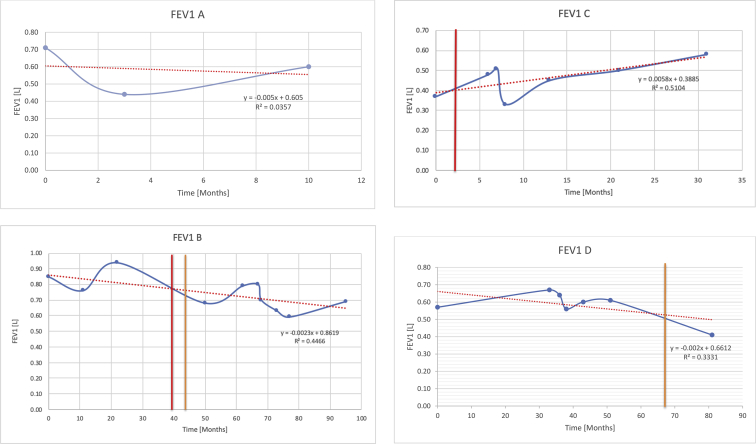

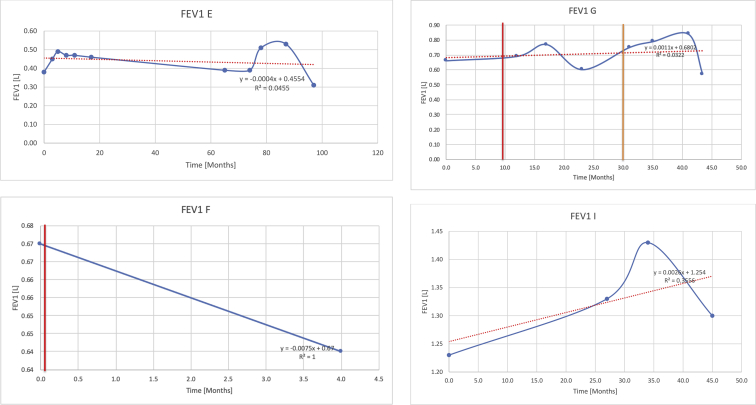

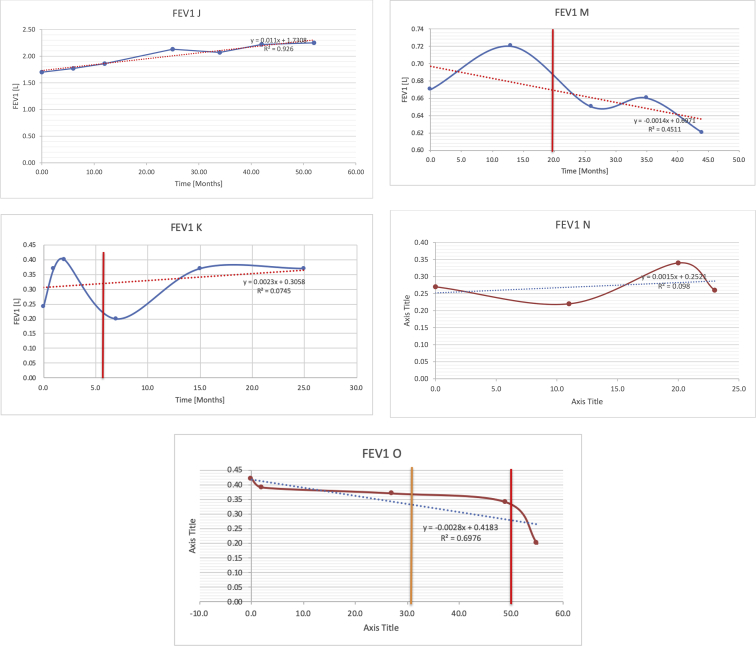
Forced expiratory volume in 1 second (FEV1) changes. The above graphs demonstrates each of the individual plots where constructed to ascertain the changes in pulmonary function over time for the given variable. The starting timepoint in the ERT group was shortly after ERT therapy had commenced. Data points are in blue and a line of best fit creating a regression line was created to ascertain the overall trend of whether there was a decline or improvement. For each of the regression curve the intercept and R^2^ value is sated under the curve. Note also, the solid red and orange bars, that mark when a therapeutic intervention was undertaken. The solid vertical, red line (**−**) indicates when adenotonsillectomy was undertaken and the orange line (**−**) illustrates when NIV was instituted. There was incomplete data for some individuals, as adenotonsillectomy was undertaken prior to formal diagnosis and thus full lung function tests would not of been undertaken at the time. Subjects A to M were ERT treated and data recorded was post commencing ERT therapy; subjects N and O were not ERT treated and data was recorded after diagnosis.

**Fig. 2:**
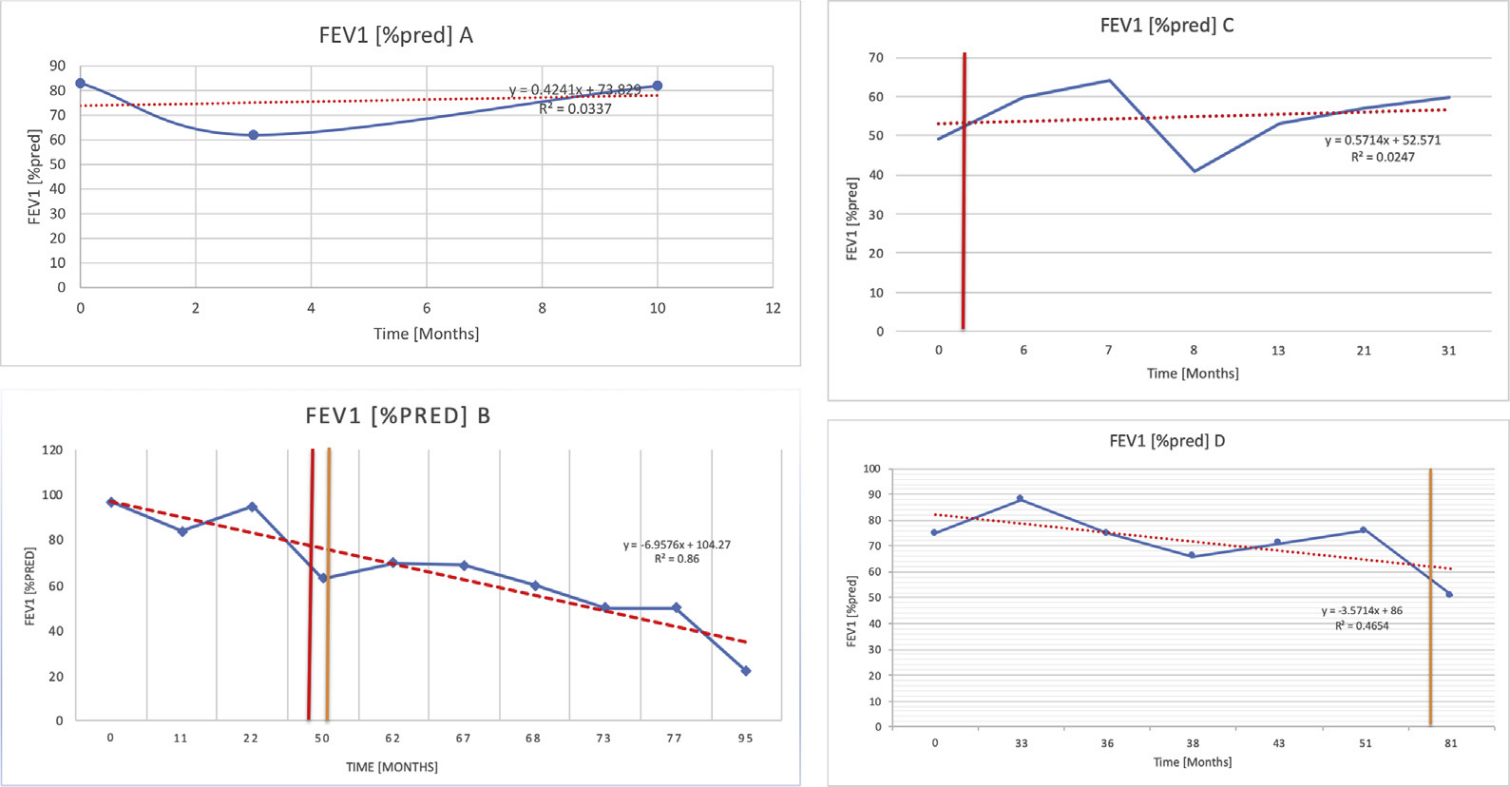

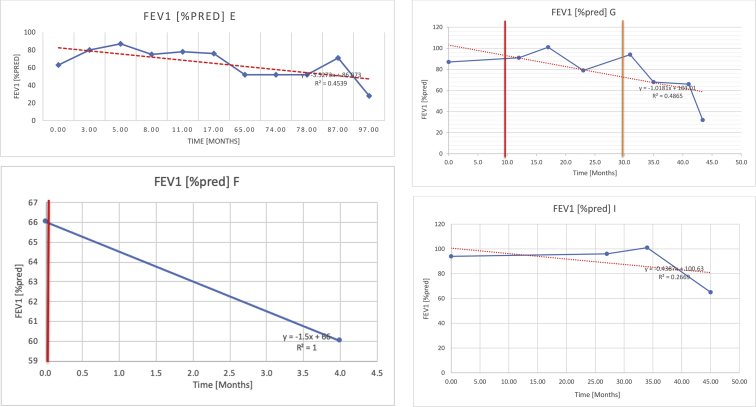

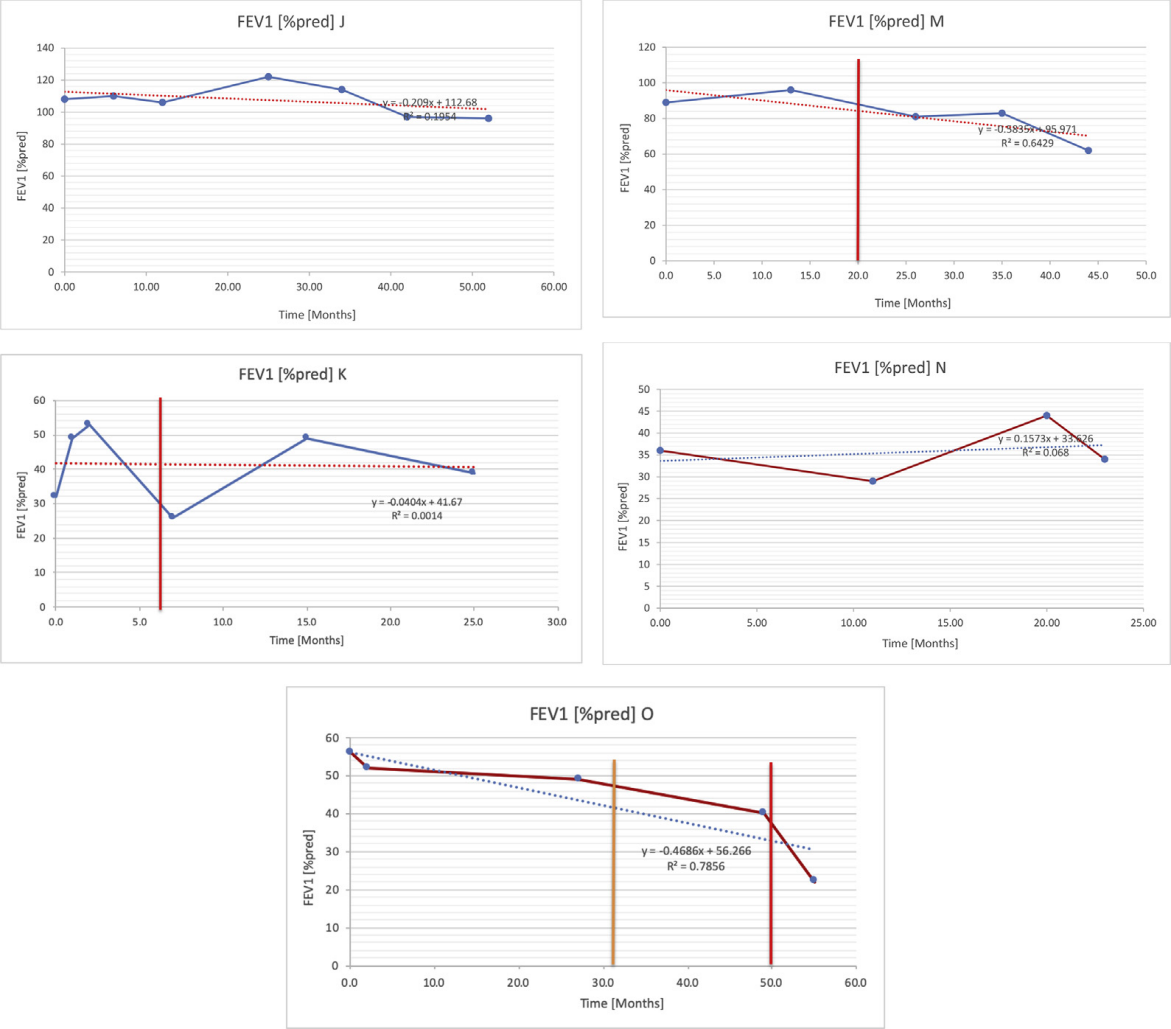
FEV1 as percentage of predicted, (FEV1 [%pred]).

**Fig. 3:**
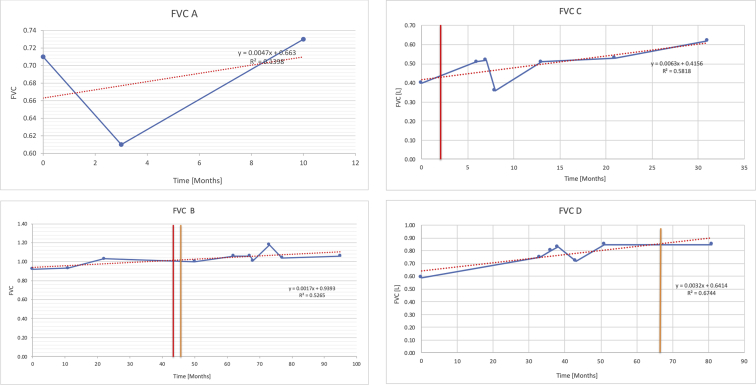

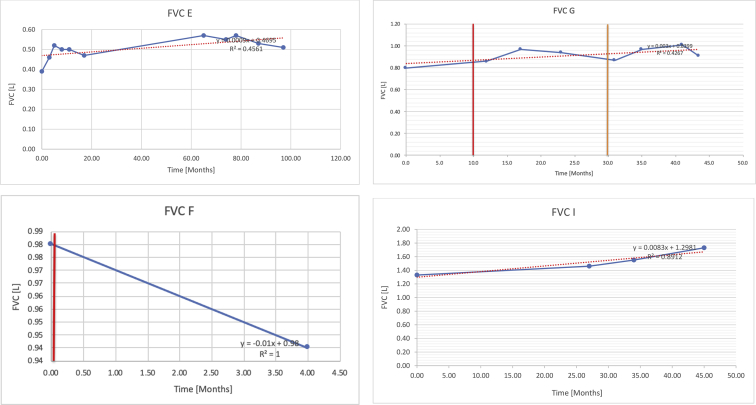

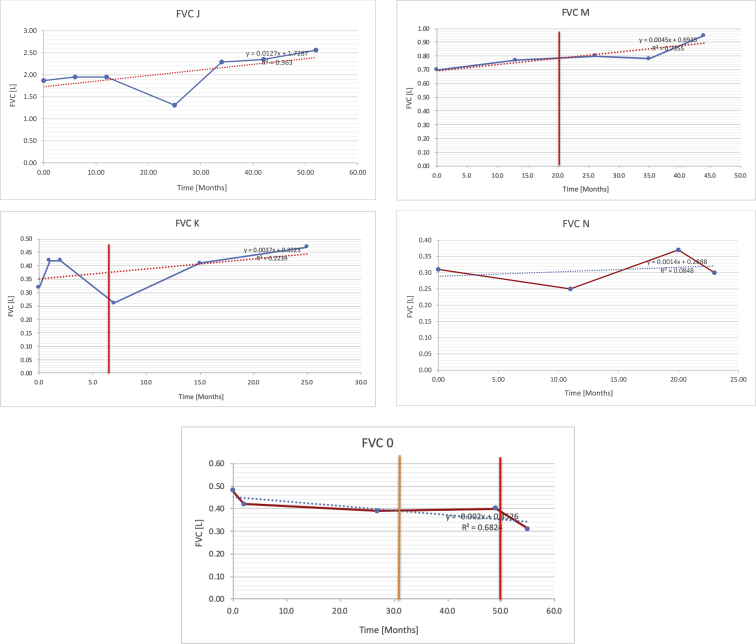
Forced vital capacity (FVC).

**Fig. 4:**
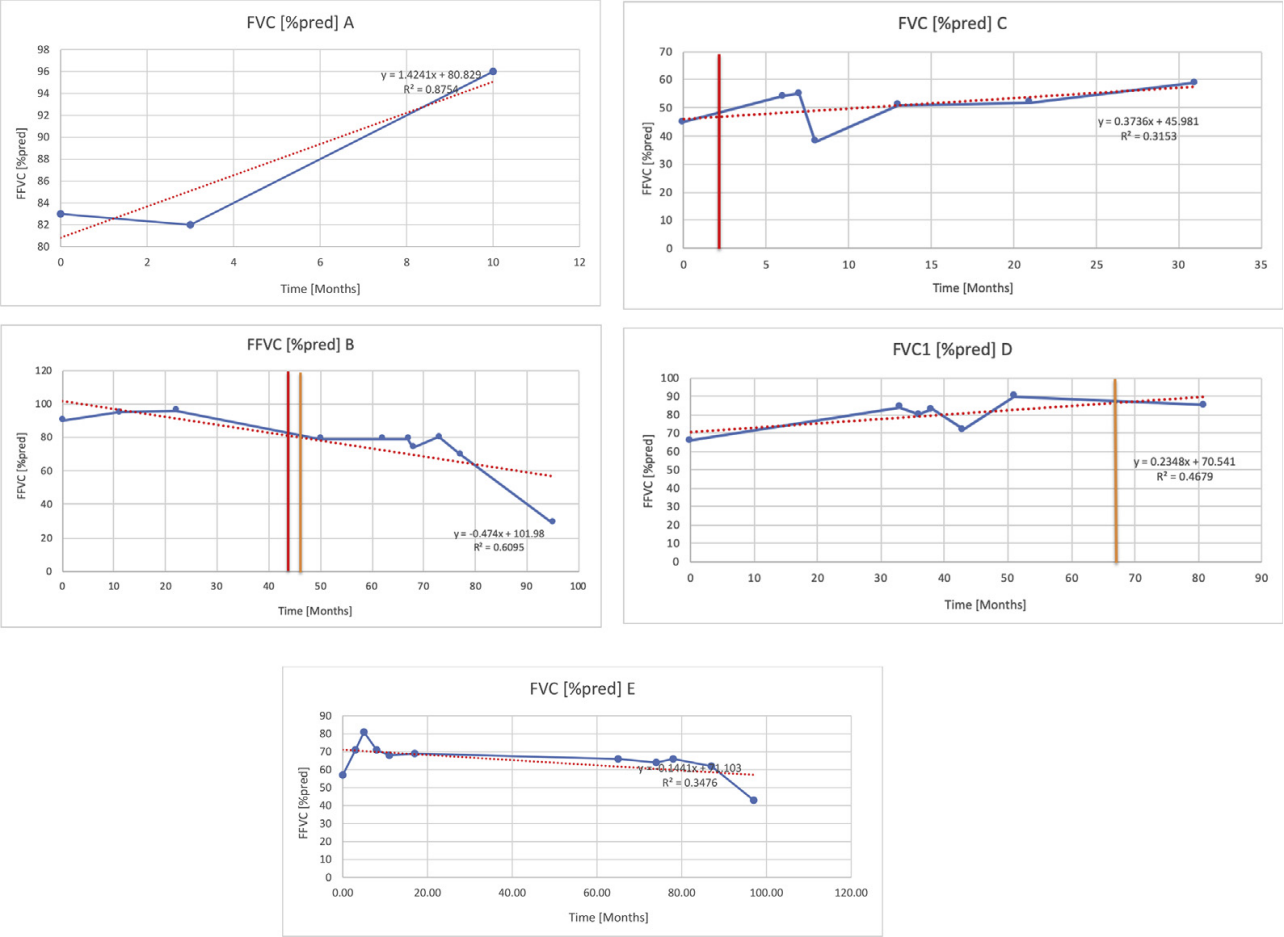

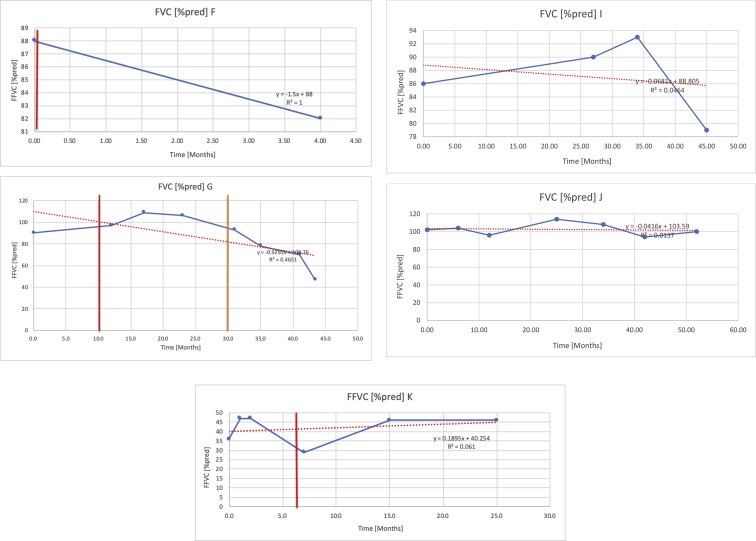

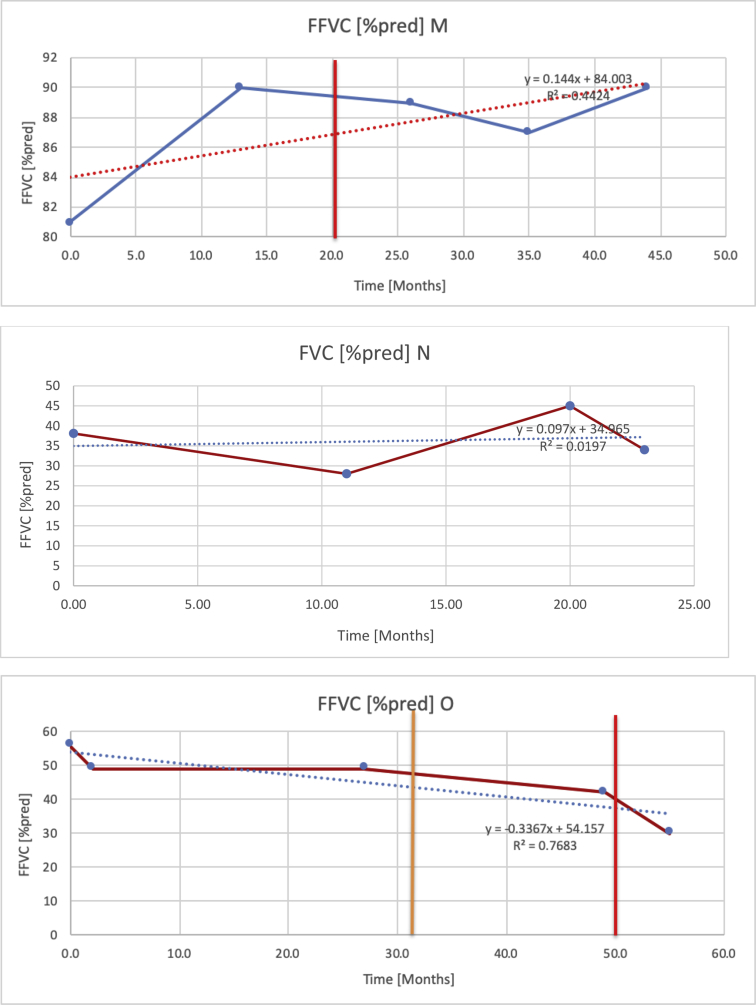
FVC [% predicted].

**Fig. 5:**
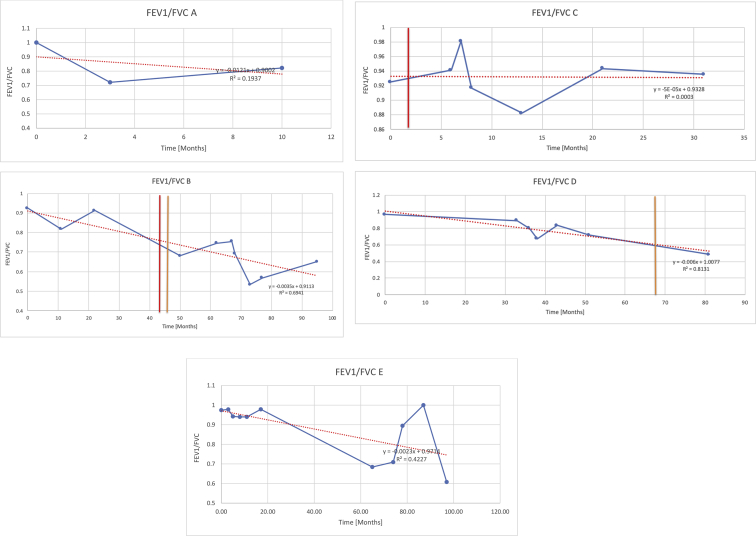

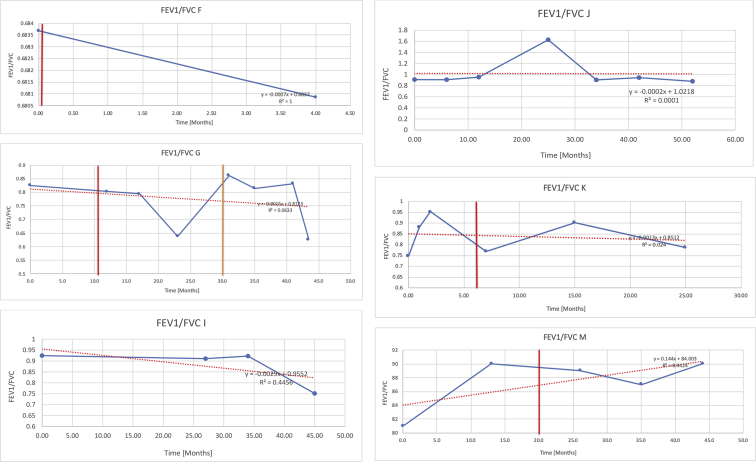

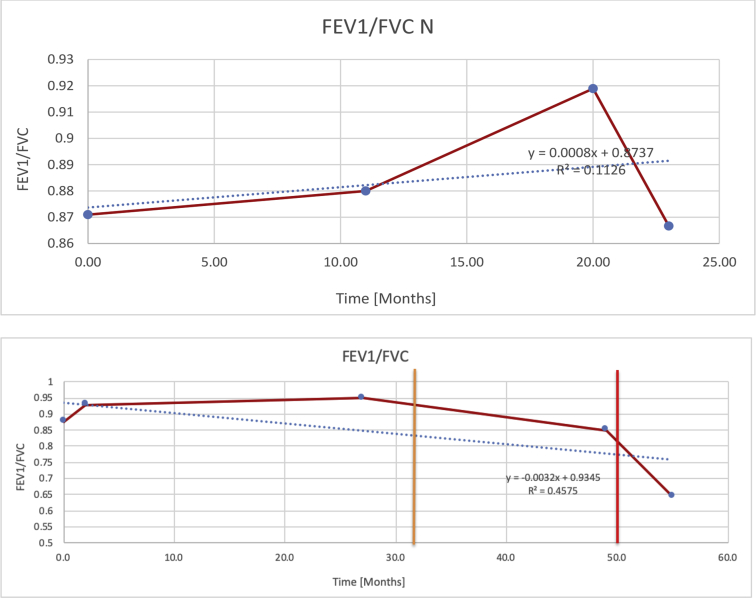
FEV1/FVC ratio.

**Fig. 6:**
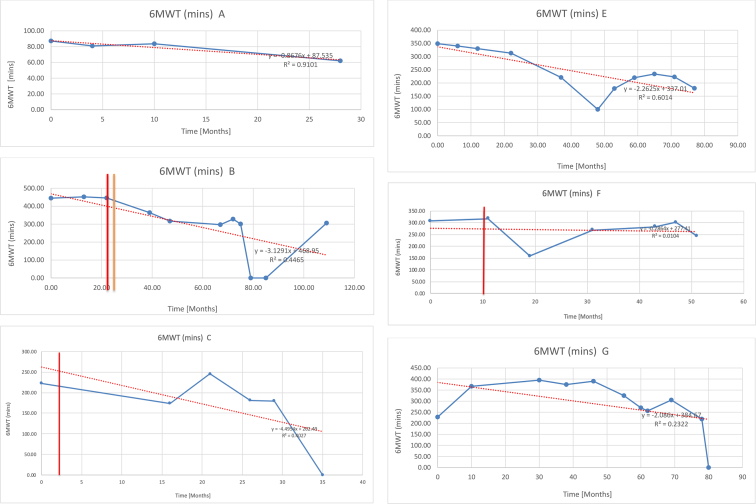

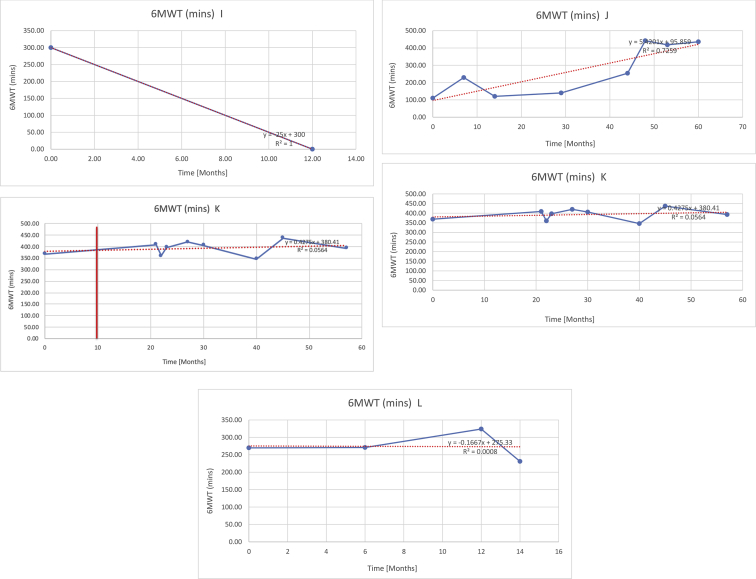
Six minute walk test (6MWT). The above graphs demonstrates each of the individual plots where constructed to ascertain the changes in pulmonary function over time for the given variable. Data points are in blue and a line of best fit creating a regression line was created to ascertain the overall trend of whether there was a decline or improvement. Note also, the solid red and orange bars, that mark when a therapeutic intervention was undertaken. The solid red line (−) indicates when adenotonsillectomy was undertaken and the orange line (−) illustrates when NIV was instituted.

Table 2 summarises the median values, range and the overall trend for the changes in pulmonary function for each of the following variables: FEV1, FEV1 [%predicted], FVC, FVC [%predicted], FEV1/FVC ratio and the six-minute walking test (6MWT). Fig. 1, Fig. 2, Fig. 3, Fig. 4, Fig. 5, Fig. 6 illustrate the data for each subject as longitudinal trajectories over time. Each graph is clearly labelled with the metric being assessed as well as the subject from which the data was obtained from. Linear regression was calculated where appropriate and the R^2^ and intercept (y) were also recorded.

**Table 2:** Summary of spirometry data.

Subject	FEV1 [Litres]	FEV1 [%pred.]	FVC [Litres]	FVC [%pred.]	FEV1:FVC	6MWT [Metres]
A	0.60 [0.44–0.71] {↓}	82 [61–83] {→}	0.71 [0.61–0.73] {↑}	83 [82–96] {↑}	0.82 [0.72–1.0] {↓}	253 [204–294 {↓}
B	0.76 [0.59–0.04] {↓}	66 [22–97] {↓}	1.04 [0.92–1.18] {↑}	79 [29–96] {↓}	0.72 [0.53–0.92] {↓}	317 [0–452] {↓}
C	0.48 [0.33–0.58] {↑}	60 [41–64] {→}	0.52 [0.36–0.62]{↑}	53 [38–59] {↑}	0.94 [0.88–0.98] {→}	180.5 [0–246] {↓}
D	0.60 [0.41–0.67] {↓}	75 [51–88] {↓}	0.8 [0.59–0.85] {↑}	83 [66–90] {↑}	0.8 [0.4–0.97] {↓}	N/A
E	0.46 [0.31–0.64] {→}	71 [28–87] {↓}	0.51 [0.39–0.57 {↑}	72 [43–90] {↓}	0.94 [0.61–0.98] {↓}	223 [100–349] {↓}
F	0.66 [0.64–0.67] {↓}	63 [60–66] {↓}	0.96 [0.94–0.98] {↓}	85 [82–88] {↓}	0.682 [0.68–0.684] {↓}	283 [159–318] {→}
G	0.72 [0.57–0.84] {→}	79 [32–101] {↓}	0.93 [0.8–1.01] {↑}	91.5 [47–109] {↓}	0.68 [0.63–0.86] {→}	305 [0–395] {↓}
I	1.32 [1.23–1.43]{↑}	95 [65–101] {↓}	1.51 [1.33–1.73] {↑}	88 [79-93 {↓}	0.91 [0.75–0.92] {↓}	150 [0–300] {↓}
J	2.07 [1.7–2.25] {↑}	108 [96–122] {→}	1.95 [1.31–2.56] {↑}	102 [94–114] {→}	0.91 [0.88–1.63] {→}	241.5 [110–442] {↑}
K	0.37 [0.2–0.4] {↑}	44 [26–53] {→}	0.42 [0.26–0.47] {↑}	46 [29–47] {↑}	0.83 [0.75–0.95] {→}	397 [346–437] {→}
M	0.66 [0.62–0.72] {↓}	83 [62–96] {↓}	0.78 [0.7–0.96 {↑}	89 [81–90] {↑}	0.85 [0.65–0.96] {↑}	N/A
N	0.27 [0.22–0.34] {↑}	35 [29-44 {↑}	0.31 [0.25–0.37] {↑}	36 [28–45] {↑}	0.88 [0.87–0.92] {↑}	N/A
O	0.37 [0.2–0.42] {↓}	49 [22–56] {↓}	0.4 [0.31–0.48] {↓}	49 [30–56] {↓}	0.88 [0.65–0.95] {↓}	N/A

Kenth et al. [1].

The table above illustrates the values for the 5 variables measured during spirometry for each subject - FEV1, Forced Expiratory Volume in the first second (Litres); FVC, Forced Vital Capacity, (Litres); as well as the six-minute walking test (6MWT). Median values are displayed in bold, minimum and maximum range are in square brackets. Curly braces {} denote the overall trends of the variable throughout the study: ↑, trend increased; ↓ trend decreased; →, no change. Subjects A-M (highlighted in yellow) were the ERT treated subjects, whilst subjects N and O (highlighted in pink) were untreated.

#### 1.3 Oximetry data

**Fig. 7:**
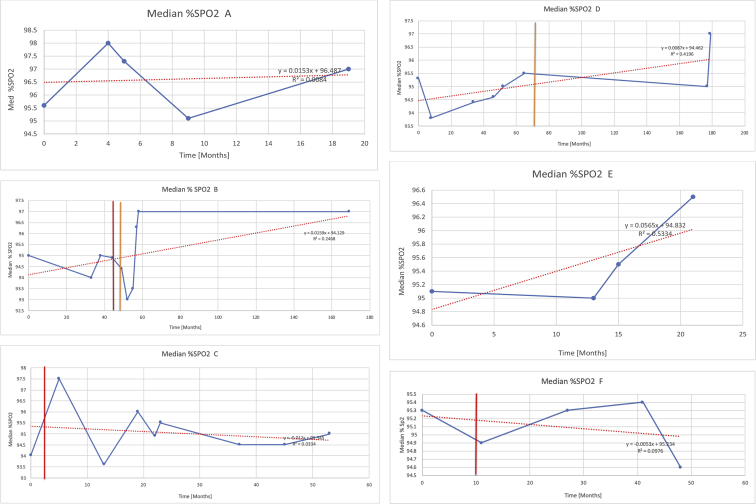

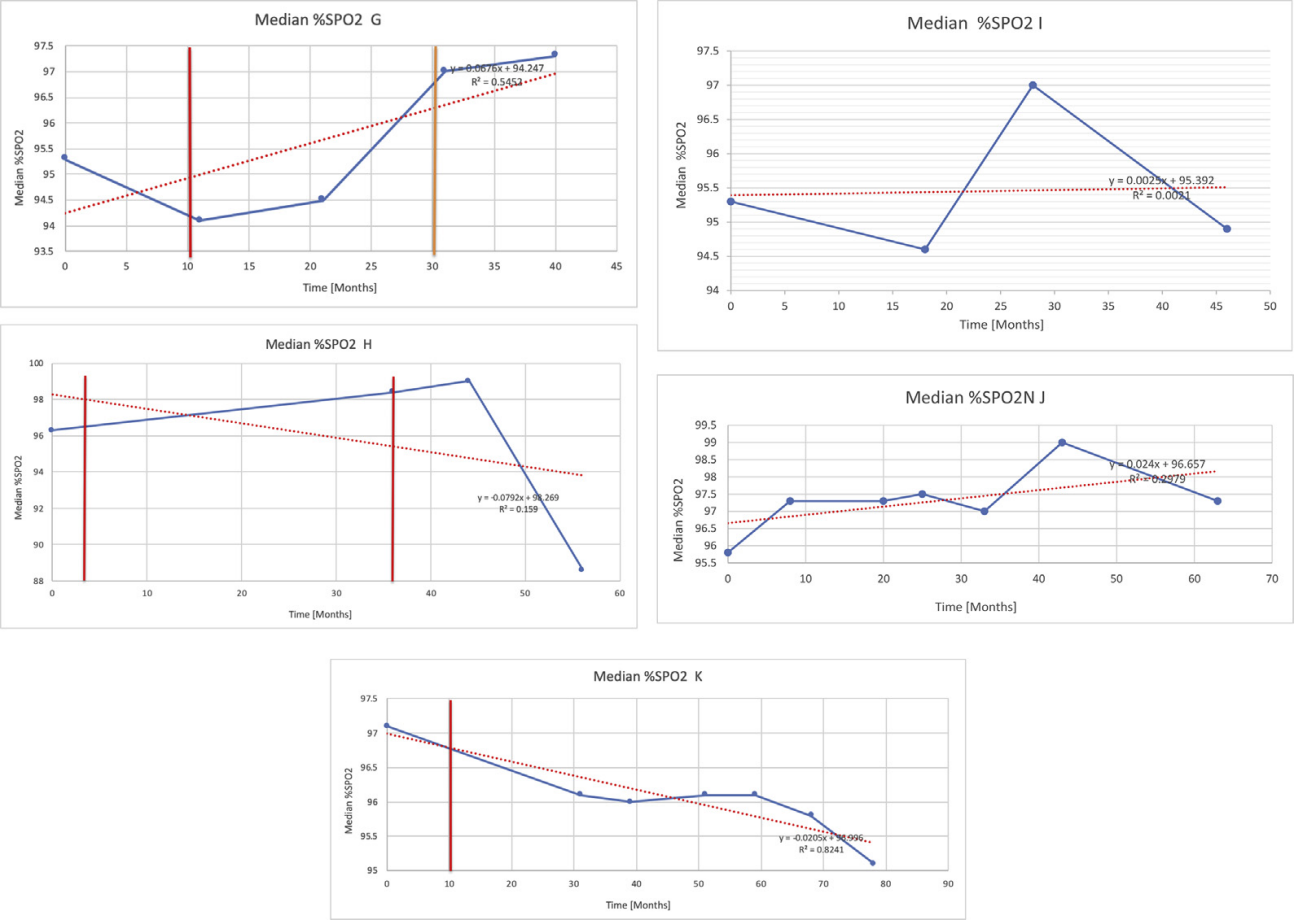

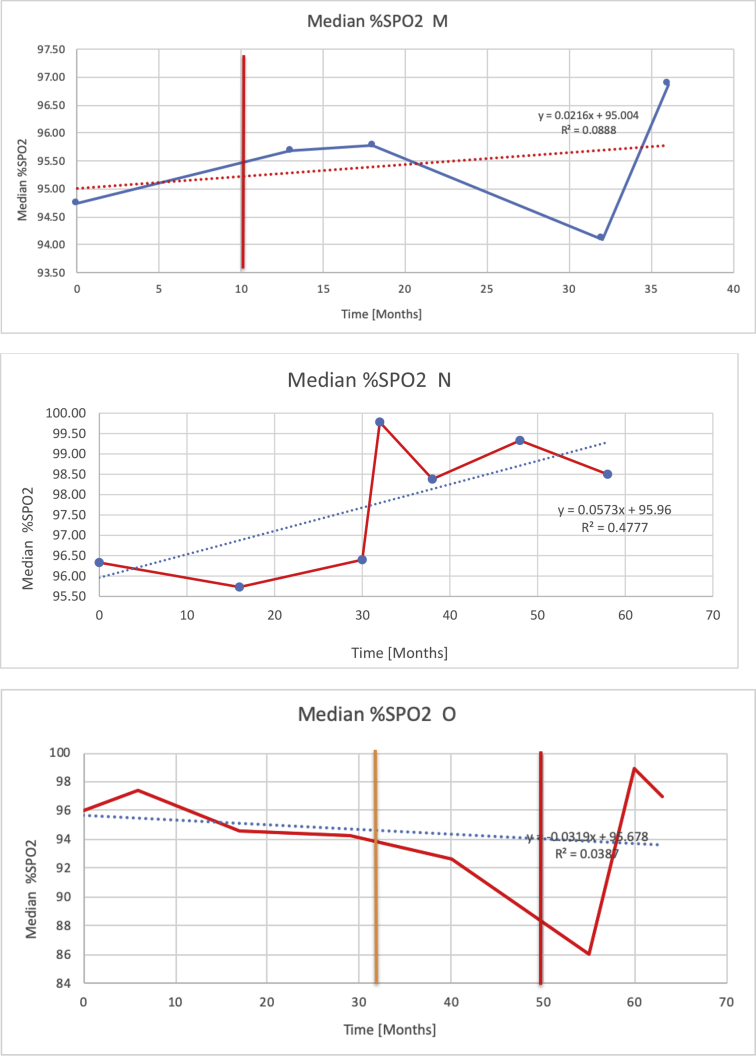
Median Spo2%.

**Fig. 8:**
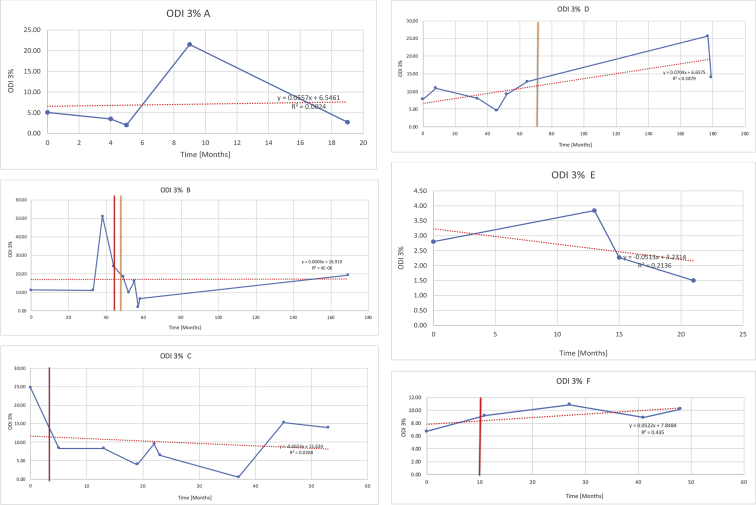

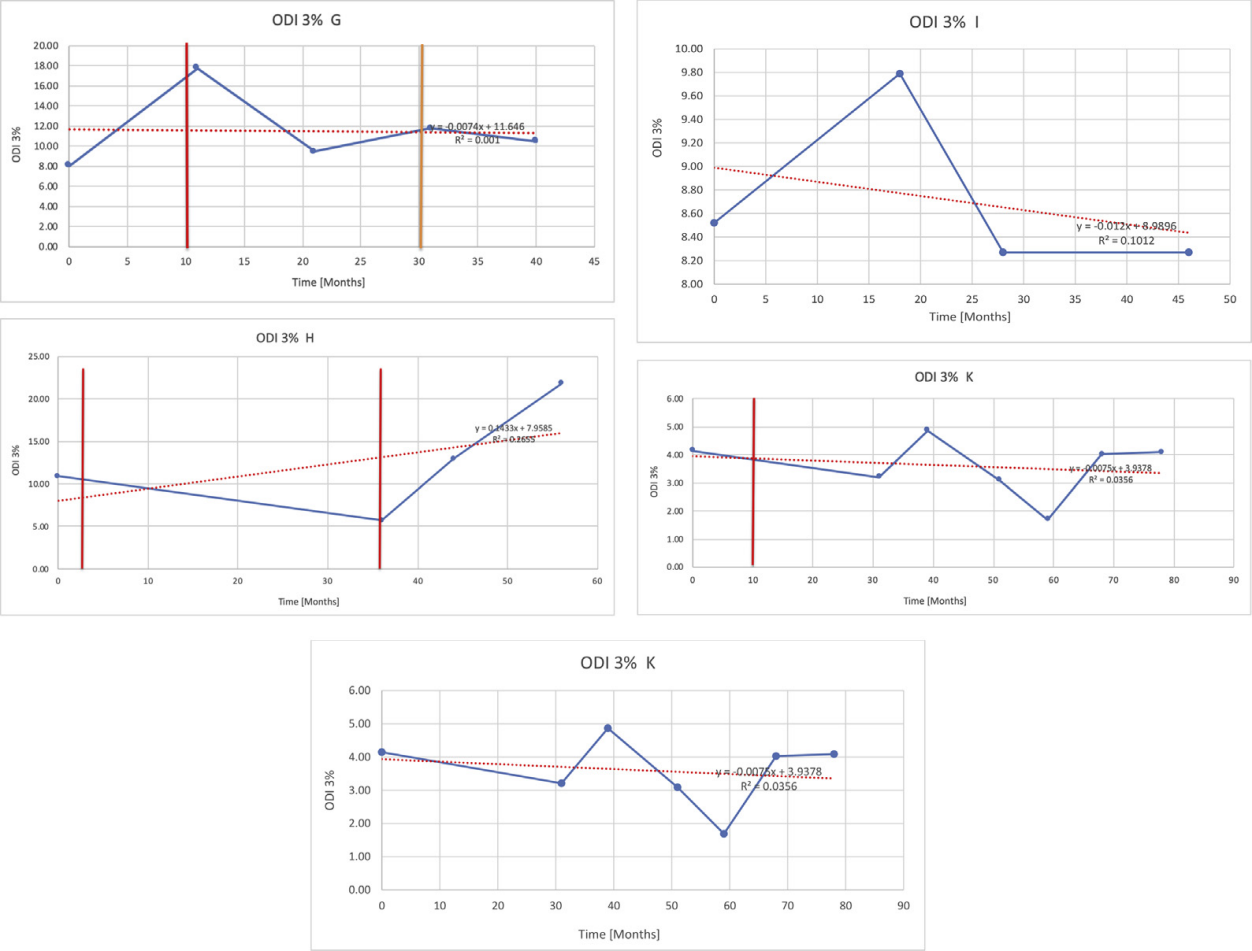

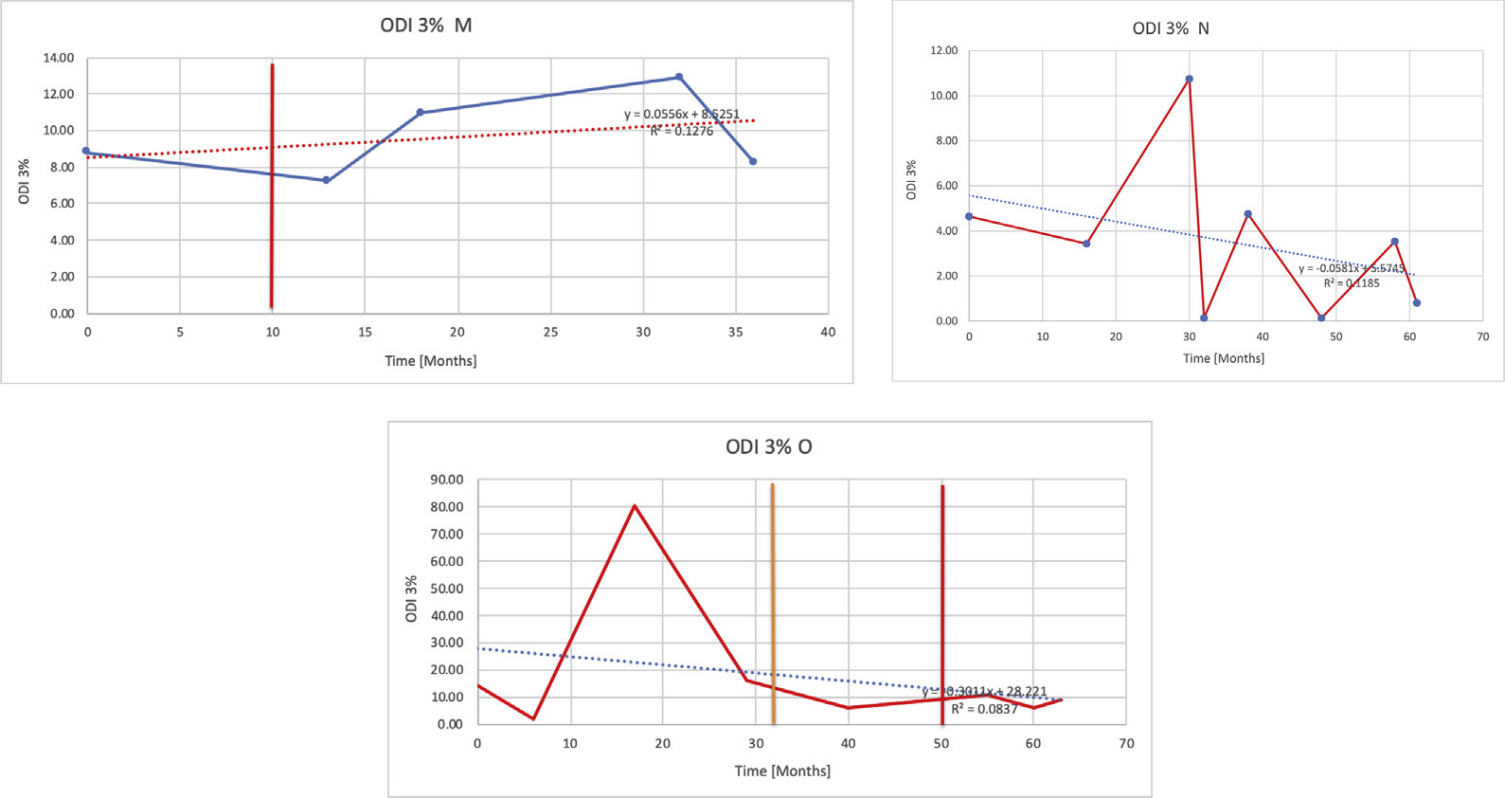
Oxygen desaturation index (ODI) 3% from baseline.

**Fig. 9:**
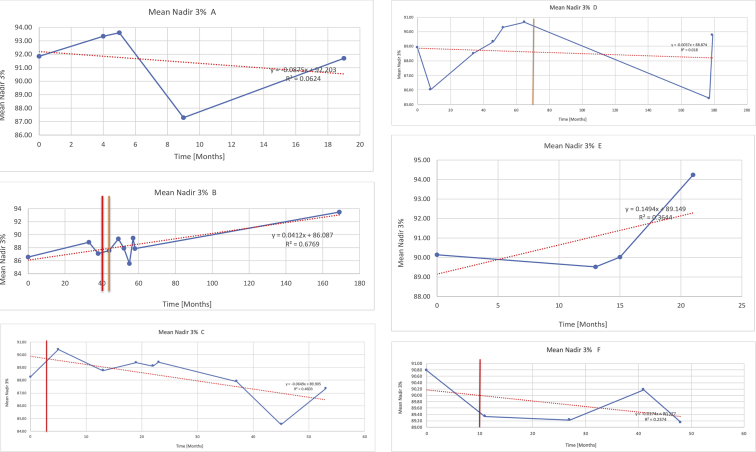

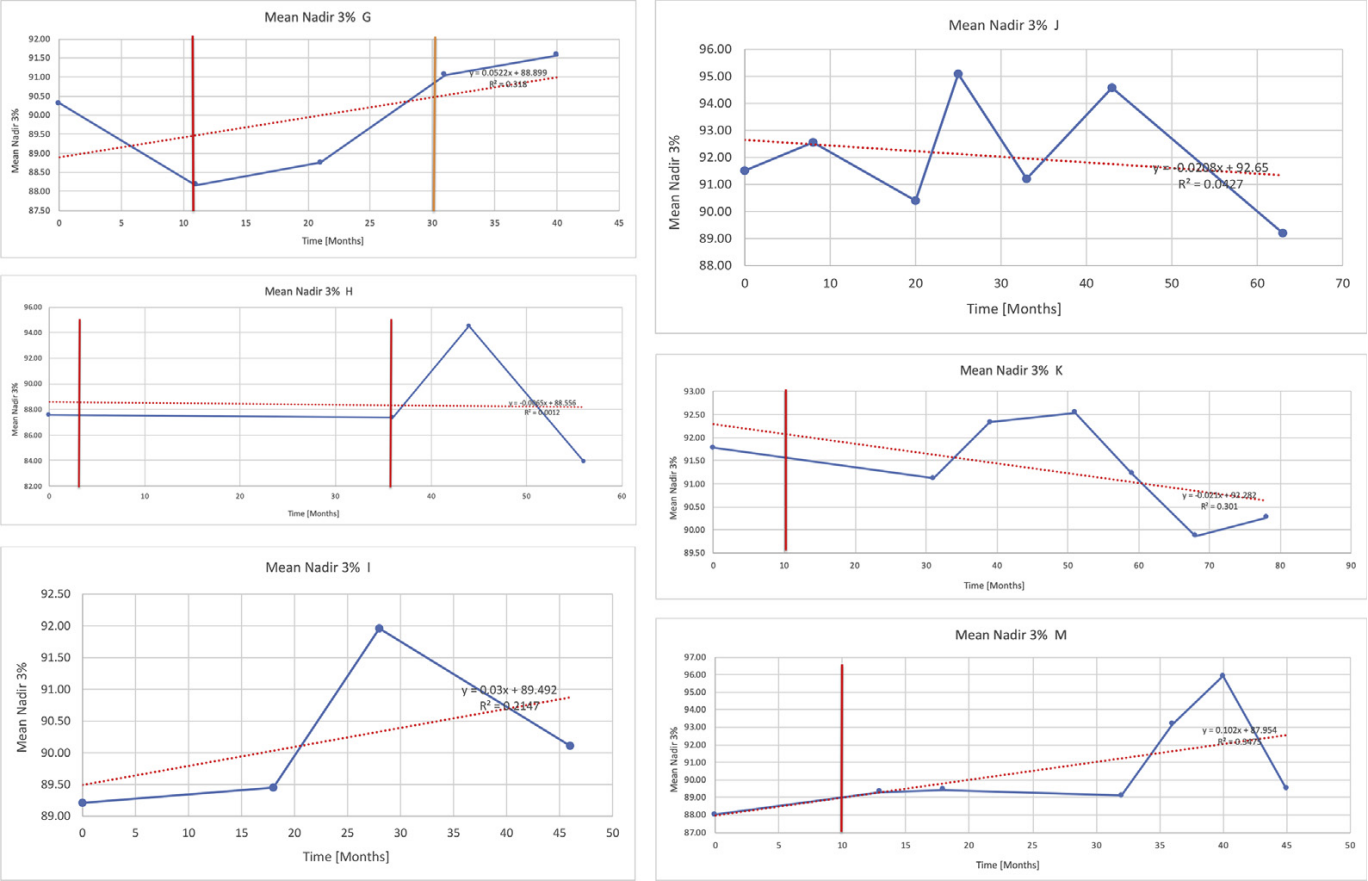

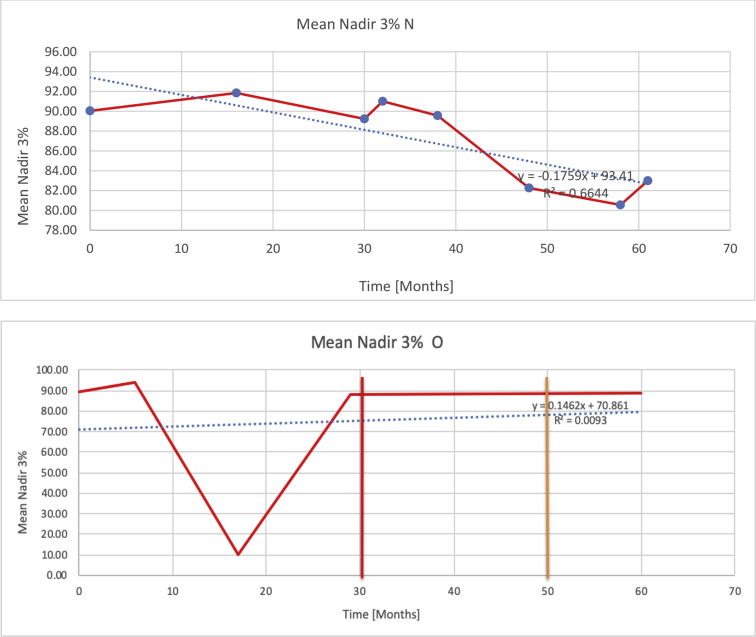
Mean nadir 3%.

**Fig. 10:**
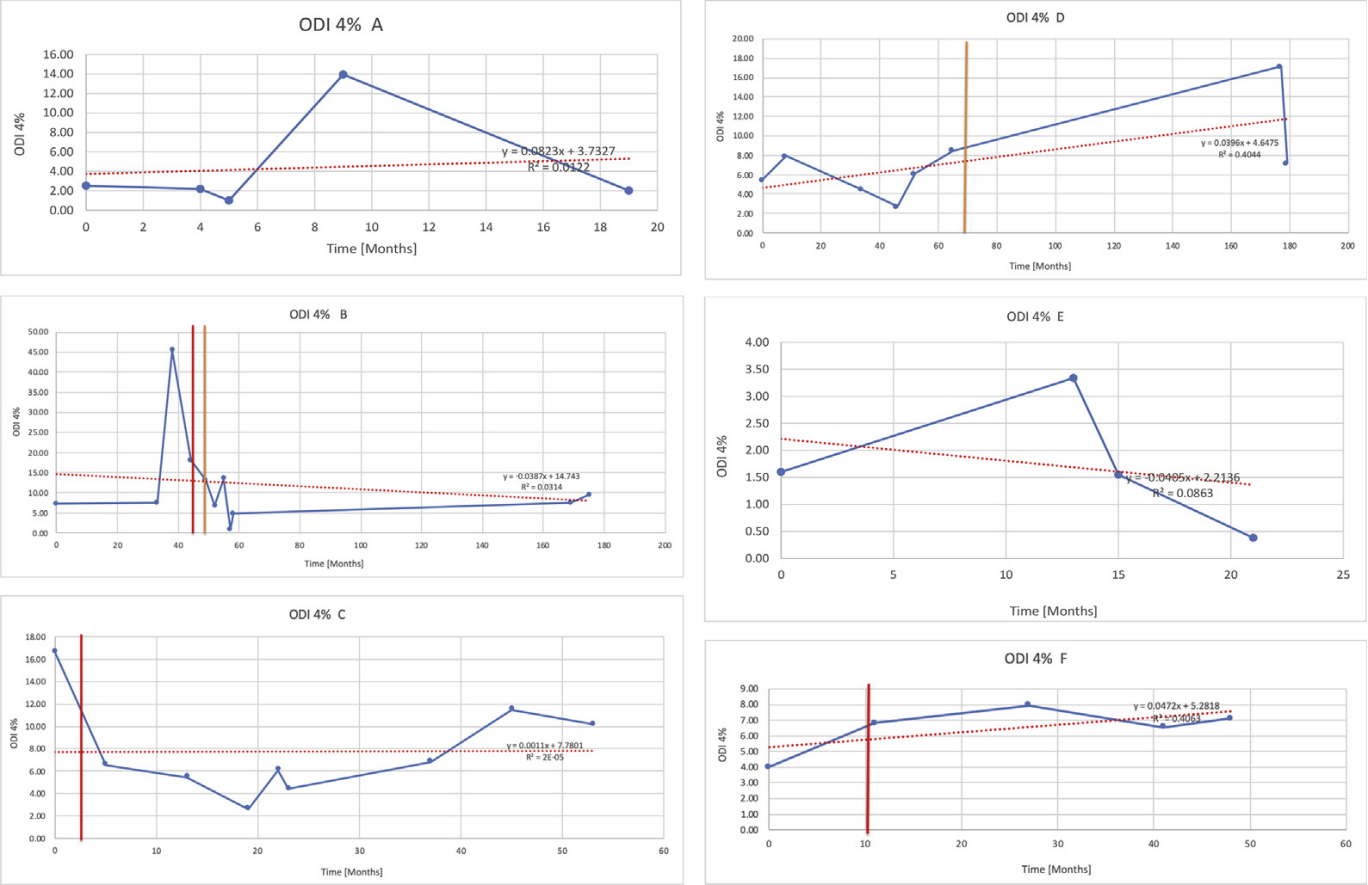

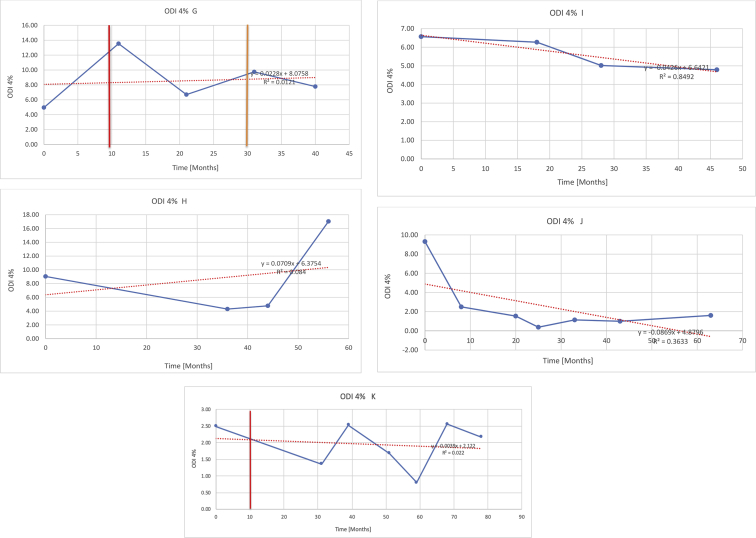

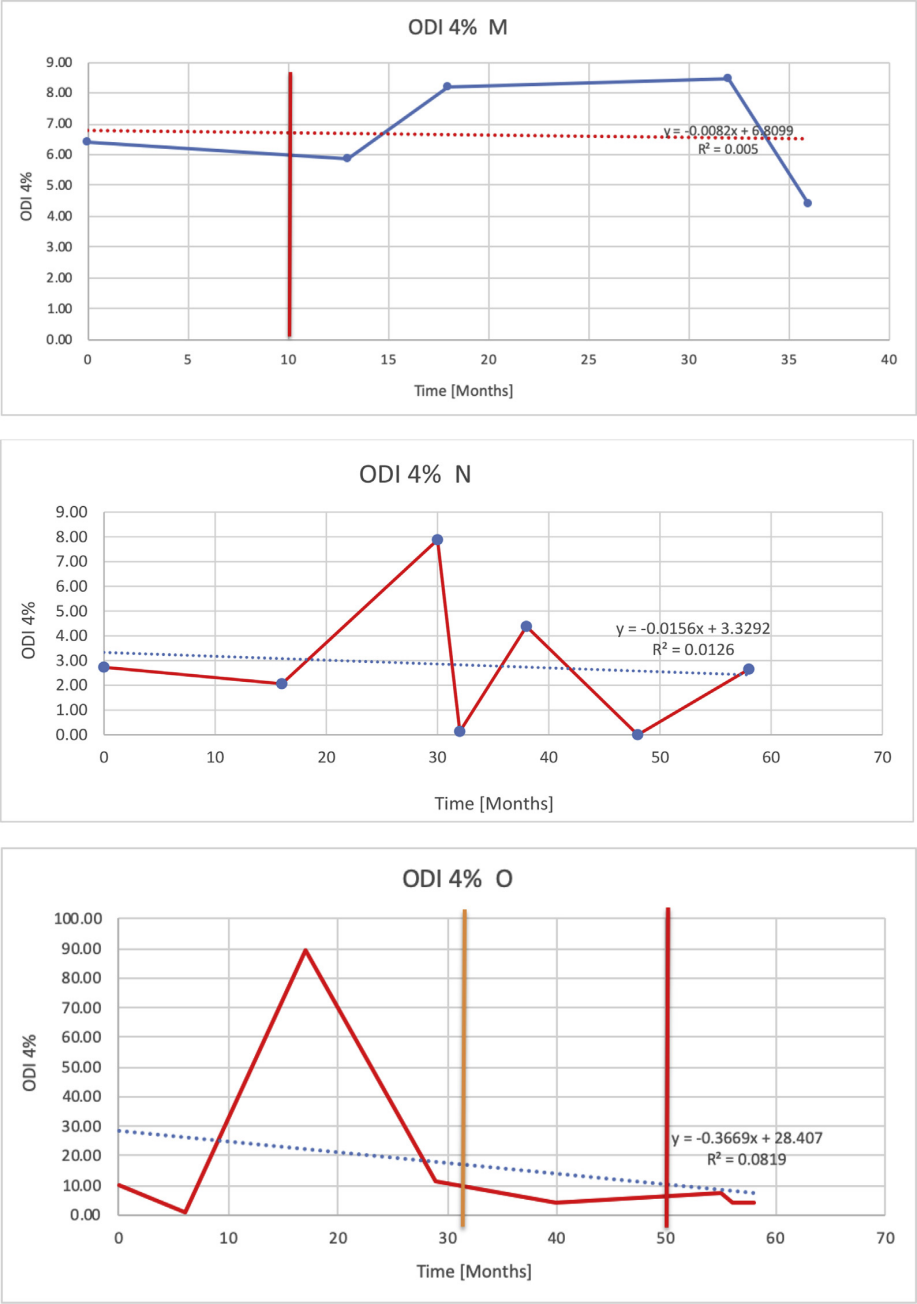
Odi 4%.

**Fig. 11:**
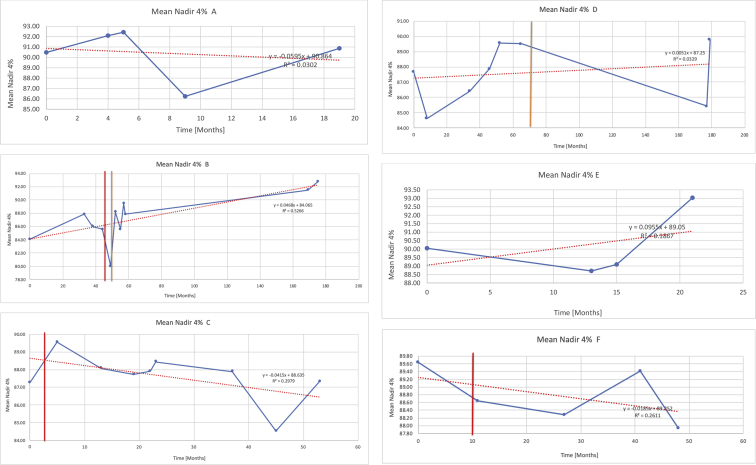

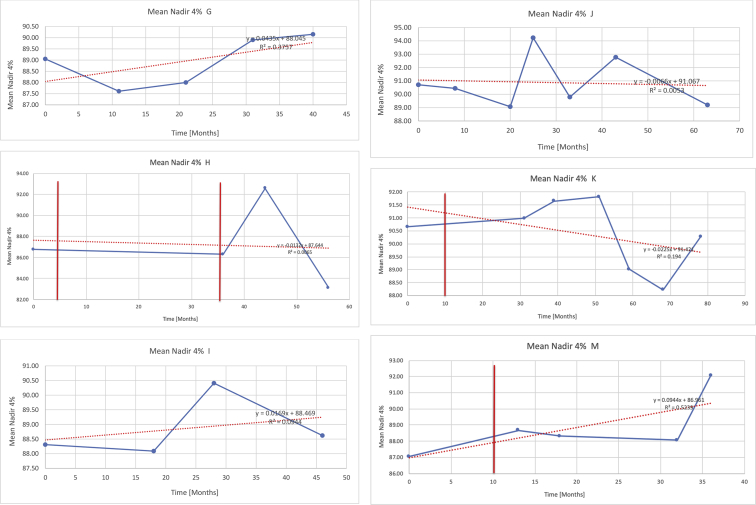

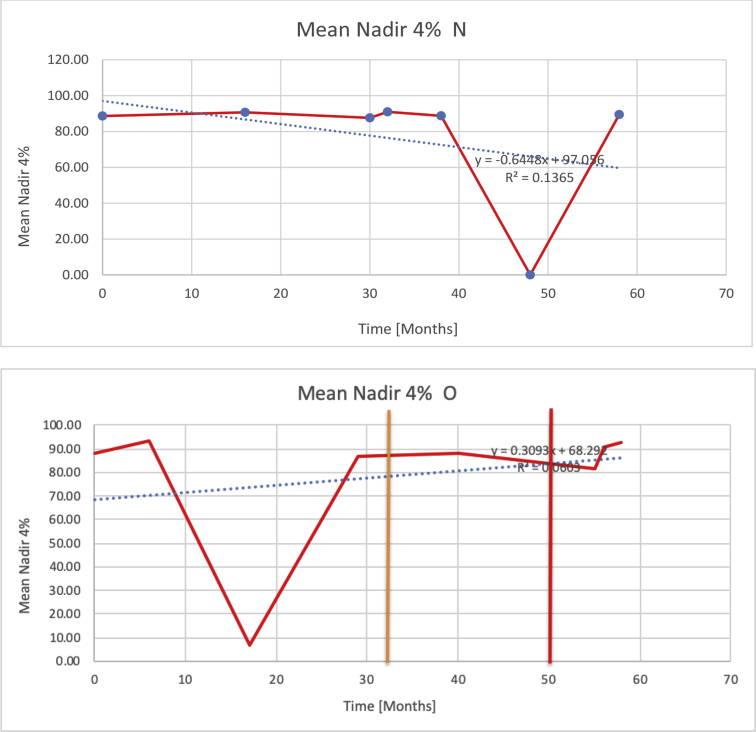
Mean nadir 4%.

**Fig. 12:**
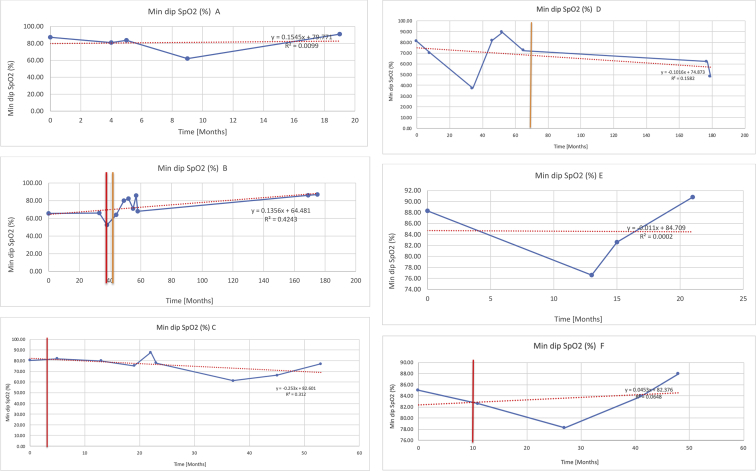

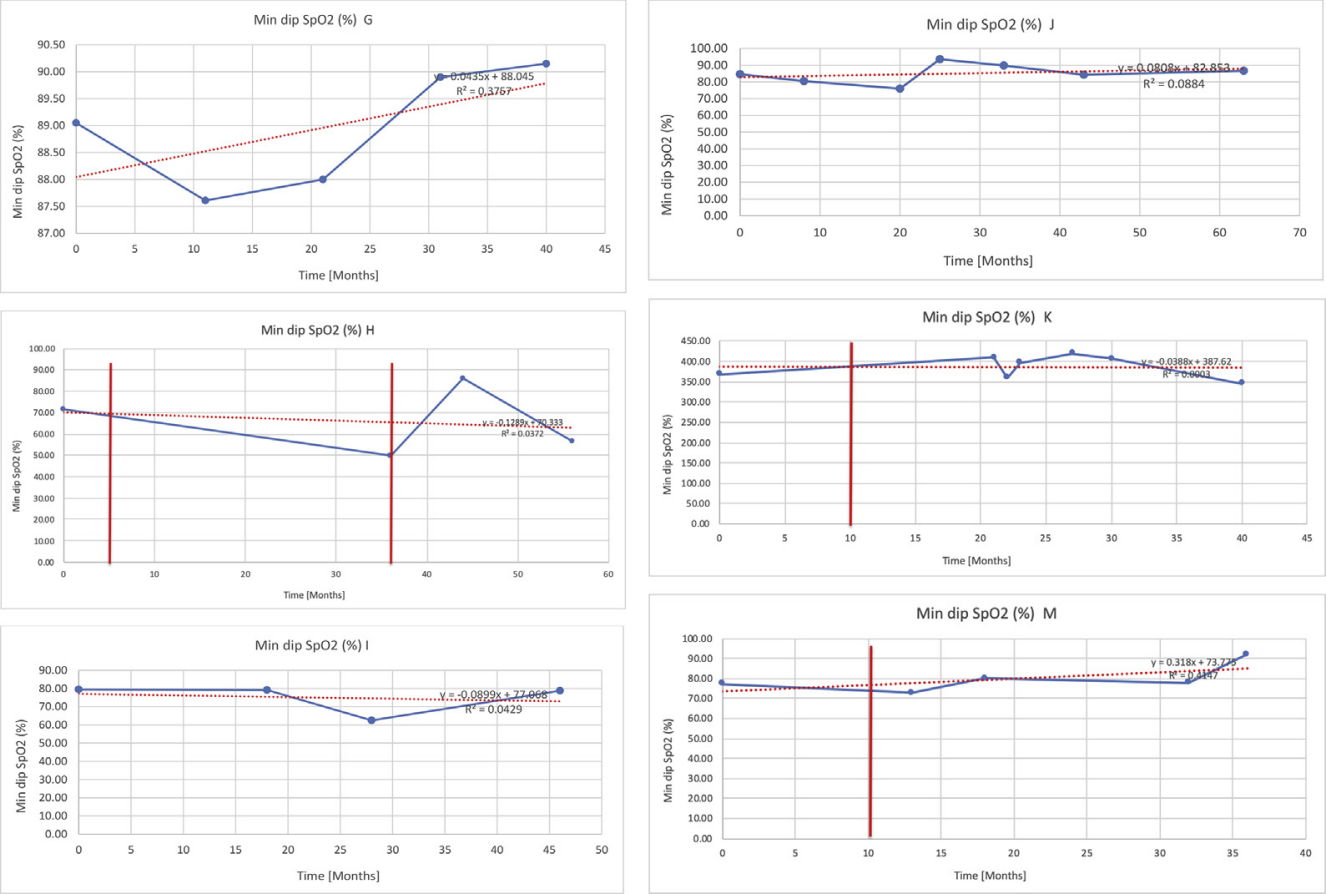

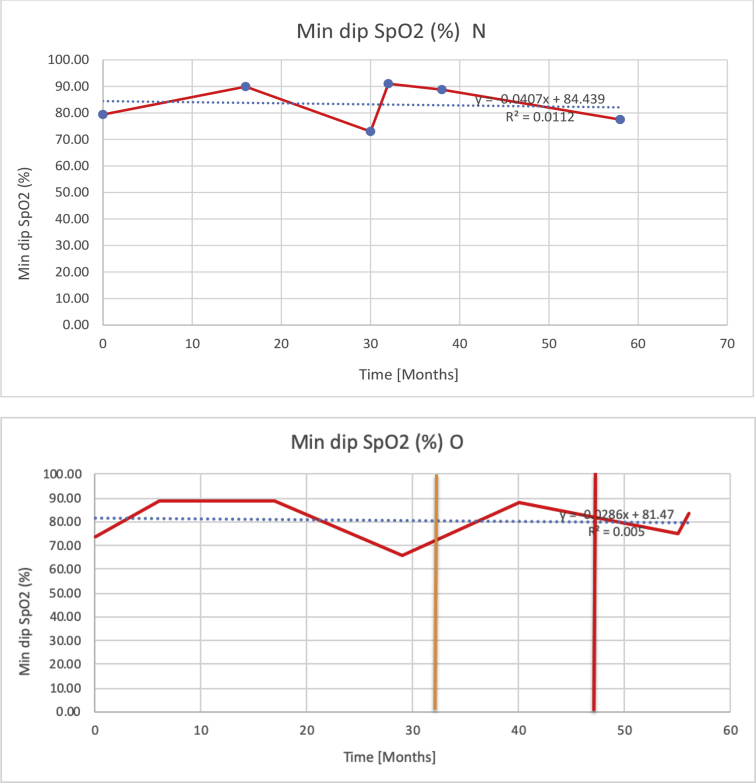
Minimum dips in % SpO2.

Table 3 illustrates the median values, range and the overall trend for the changes in oximetry. These included data for median %Spo2, ODI 3%, mean nadir 3%, ODI 4%, mean nadir 4% and min dip SpO2 (%). Fig. 7, Fig. 8, Fig. 9, Fig. 10, Fig. 11, Fig. 12 illustrate the individual plots for each subject (labelled) over time. As above, linear regression was calculated where appropriate and the R^2^ and intercept (y) were also recorded.

**Table 3:** Summary of oximetry results.

Subject	Oximetry Variable					
	Median %Spo2	ODI 3%	Mean Nadir 3%	ODI 4%	Mean Nadir 4%	Min dip. SpO2 (%)
A	97 [95.1–98] {→}	3.5 [2–21.5] {→}	91.9 [87.3–93.6] {↓}	2.2 [1–13.9] {→}	90.9 [86.2–92.4] {↓}	83.6 [62–90.9] {→}
B	95 [93–97] {↑}	13.6 [2–51.2] {→}	87.8 [85.6–93.5] {↑}	7.5 [0.9–45.4] {→}	87.8 [80–92.8] {↑}	71 [52.5–87] {↑}
C	94.9 [93.6–97.5] {→}	8.3 [0.4–24.6] {↓}	88.8 [84.5–90.4] {↓}	6.5 [2.6–16.7] {→}	87.9 [84.5–89.6] {↓}	78 [61.4–87.9] {↓}
D	95 [93.8–97] {↑}	10 [4.6–25.7] {↑}	89.1 [85.4–90.7] {→}	6.6 [2.7–17.2] {↑}	87.8 [84.6–89.8] {↑}	71.3 [37.1–89.6] {↓}
E	95.3 [95–96.5] {↑}	2.5 [1.5–3.8] {↓}	90.8 [89.5–94.3] {↑}	1.6 [0.4–3.3] {↓}	89.6 [87.6–90.3] {↑}	85.5 [76.6–90.8] {→}
F	95.3 [94.6–95.4] {→}	9.2 [6.7–10.9] {↑}	89.3 [89.2–90.8] {↓}	6.8 [4–8] {↑}	88.6 [87.9–89.6] {↓}	83.9 [78.2–87.9] {↑}
G	95.3 [94.1–97.3] {↑}	10.5 [8–17.8] {↓}	90.3 [88.2–91.6] {↑}	7.8 [5–13.5] {→}	89.1 [87.6–90.2] {↑}	77.8 [82.9–90.2] {↑}
H	97.4 [88.6–99] {↓}	11.9 [5.7–21.8] {↑}	87.5 [83.9–94.5] {→}	6.9 [4.3–17] {↑}	86.6 [83.1–92.6] {→}	64 [49.9–86] {→}
I	95.1 [94.6–97] {→}	8.4 [8.3–9.8] {↓}	89.8 [89.2–92] {↑}	5.6 [4.6–6.6] {→}	88.5 [88.1–90.4] {→}	79 [62.5–79.5] {→}
J	97.3 [95.8–99] {↑}	2 [0.9–16.4] {↓}	90.1 [89.2–94.6] {→}	1.5 [0.4–9.3] {↓}	90.4 [89.1–94.3] {→}	84.6 [76–93.6] {↑}
K	96.1 [95.1–97.1] {↓}	4 [1.7–4.9] {↓}	91.2 [89.9–92.5] {↓}	2.2 [0.8–2.6] {→}	90.7 [88.2–91.8] {↓}	87.8 [73.2–89.1] {→}
M	95.9 [94.3–97] {→}	8.8 [7.2–12.9] {→}	89.3 [88–93.2] {↑}	6.4 [4.4–8.4] {→}	88.3 [87–92.1] {↑}	78 [72.8–92.1] {↑}
N	98.6 [96–100] {→}	3.5 [0.2–10.8] {↓}	90.4 [82.3–91.9] {↓}	2.7 [0–7.9] {→}	89.1 [87.6–91] {↓}	84.1 [73–91] {→}
O	94.6 [86–98.9] {↓}	9.9 [2–80.5] {↓}	89.3 [88–94.4] {→}	5.8 [0.8–89.6] {↓}	88.1 [6.58–93.4] {↑}	83.5 [66–89.2] {→}

Kenth et al. [1].

The table above illustrates the values for the 6 variables measured during oximetry testing for each subject. Median values are displayed in bold, minimum and maximum range are in square brackets. Curly braces {} denote the overall trends of the variable throughout the study: ↑, trend increased; ↓ trend decreased; →, no change in trend. ODI 3%, ≥3% arterial oxygen desaturations/hour; ODI 4%, ≥4% arterial oxygen desaturations/hour; min dip SpO2, minimum dips in oxygen saturations. Subjects A-M (highlighted in yellow) were the ERT treated subjects, whilst subjects N and O (highlighted in pink) were untreated.

### 2 Experimental design, materials, and methods

MPS IVA is associated with severe, debilitating airway and respiratory disease [1,2]. The methodology employed to assess pulmonary function in this study is validated by and aligned to The American Thoracic Society [3,4] and the British Thoracic Society guidance. The methodology used for oximetry as previously described by Pal et al. (2015) [5], based on the American Academy of Sleep Medicine (AASM) manual for the scoring of sleep and associated events [5,6]. Composite clinical endpoints used in this study for evaluating pulmonary function included spirometry variables (FEV1, FEV1 [%Pred] FVC, FVC [%Pred], FEV1/FVC); sleep studies oximetry variables (median %Spo2, ODI 3%, mean nadir 3%, ODI 4%, mean nadir 4% and min dip SpO2 (%)) and 6MWT for cardiorespiratory reserve.

MPS IVA patients treated at the Royal Manchester Children's Hospital, Manchester (UK) between 2009 and 2018, were identified from an existing patient database and medical records. This yielded a study group of 16 subjects for whom long-term follow-up was available at a single centre. A retrospective review was undertaken of baseline demographics, spirometry and oximetry (sleep studies), ERT and other therapeutic interventions – including both medical and surgical measures. The data for each subject was tabulated and examined sequentially over the study period to provide a nuanced characterization of the changes in pulmonary function, evolution and natural history of disease progression. The subjects in this study included those from the MOR 100 phase 1 study (n = 5), MOR 005 phase III placebo-controlled (n = 5) and the MOR 007 trial (n = 6) [7].
